# Lubricity of Ethanol–Diesel Fuel Blends—Study with the Four-Ball Machine Method

**DOI:** 10.3390/ma14102492

**Published:** 2021-05-12

**Authors:** Hubert Kuszewski, Artur Jaworski, Maksymilian Mądziel

**Affiliations:** Faculty of Mechanical Engineering and Aeronautics, Rzeszow University of Technology, 35-959 Rzeszów, Poland; hkuszews@prz.edu.pl (H.K.); mmadziel@prz.edu.pl (M.M.)

**Keywords:** fuels, ethanol, lubrication, diesel engine, tribology

## Abstract

Due to the increasing consumption of fuels in heavy industries, especially in road transportation, significant efforts are being made to increase the market participation of renewable fuels, including ethanol. In diesel engines, however, ethanol cannot be used as a pure fuel, primarily due to its very low cetane number and lubricity. For this reason, greater attention is being paid to blended fuels containing diesel and varying percentages of ethanol. Tests of lubricating properties carried out in accordance with the standard HFRR (high frequency reciprocating rig) method for ethanol–diesel fuel blends have long durations, which leads to ethanol evaporation and changes in the composition of the tested fuel sample under elevated temperatures. Therefore, this study presents an alternative lubricity assessment criterion based on the measurement of the scuffing load with a four-ball machine. Lubricity tests of blends of typical diesel fuel and ethanol, with ethanol volume fractions up to 14% (*v*/*v*), were conducted using a four-ball machine with a continuous increase of the load force of the friction node. In this method the lubrication criterion was the scuffing load of the tribosystem. The obtained results provided insights into the influence of the addition of ethanol to diesel fuel on lubricating properties, while limiting the ethanol evaporation process. The results also showed that an increase in the fraction of ethanol up to 14% (*v*/*v*) in diesel fuel resulted in a decrease in the scuffing load and a corresponding deterioration in the lubricating properties of the diesel–ethanol blend. For an ethanol volume fraction of 6–14%, the changes in the scuffing load were smaller than in ethanol volume fractions of 0–6%.

## 1. Introduction

Internal combustion engine systems include both moving and stationary parts; one such part is a reciprocating piston inside the engine cylinder. During engine operation, the friction forces that are generated cause wear on the contact of the sliding elements. This contributes to the deterioration of the reliability parameter, which directly relates to operating costs [1,2]. A solution to this problem is to properly lubricate the engine parts. Therefore, it is important to use a fuel with good lubricity to improve the overall durability of the engine [3].

One of the most important parameters for precision pairs of compression-ignition-engine supply systems is the lubricity of the fuel [4,5]. To counteract the phenomenon of the seizing up of the friction connections in the engine supply system, the friction surfaces must be separated by a durable layer of lubricant. Most injection systems in compression-ignition engines utilize the lubricant as the fuel. A drawback of this approach is that boundary lubrication can occur, whereas hydrodynamic lubrication [6] is more desirable. This result is significant because fuels that have better lubricating properties are also better at creating permanent boundary layers.

Environmental regulations targeting exhaust gas emissions [7,8], as well as the relatively high prices of fuels derived from the processing of crude oil [9,10], have led to increased interest in alternative fuels [11]. Simultaneously, the use of diesel engines in industry and transport, especially on the road, continues to grow [12,13,14]. For decades, vegetable oil esters have been added to diesel fuel in these types of engines; more recently, there has been increased interest in using various alcohols as fuel additives [15,16,17,18,19]. Ethanol is a particular focus in this area of research because it can be produced from plants and can therefore be considered a fully renewable fuel [19,20,21,22,23].

However, due primarily to its very low cetane number, i.e., a very low propensity for self-ignition, as shown in [23,24,25,26,27,28], ethanol cannot be used as a pure fuel in diesel engines. For this reason, blending diesel fuel with ethanol is considered a viable option, especially given the fact that dehydrated ethanol has been shown to have relatively good miscibility with diesel fuel [24,29,30]. Blends with higher percentages of ethanol in diesel fuel require significant modifications to adapt their parameters to the requirements of typical diesel fuels; therefore, studies are mostly focused on diesel–ethanol blends containing up to 15% (*v*/*v*) ethanol [23].

Fuel lubrication is essential for the durability of the precision pairs of diesel engine injection systems [4,31]. In order to prevent the seizing up of the tribosystem in the fuel system, the frictional surfaces must be separated by a durable layer of lubricant.

Assessment of the tribological properties of fuels requires the use of appropriate test methods, including laboratory tests, bench tests conducted directly using fuel supply system elements, and operational tests after a specified vehicle mileage or engine operational time. Many test methods are used to assess the lubricating properties of fuels under laboratory conditions; the most well-known of which are the HFRR, Lucas dwell test, Lucas four-ball test, Thornton aviation fuel lubricity evaluator (TAFLE), ball on three seats (BOTS), ball on three discs (BOTD), ball on cylinder lubricity evaluator (BOCLE), scuffing load BOCLE (SLBOCLE), and Cameron–Plint test (roller on plate) [5,32,33,34]. Alternatively, a four-ball machine can be used to evaluate the lubricating properties [35,36,37]. Particularly in [36], the authors used a four-ball tribo-tester to measure wear and friction characteristics of some biodiesel samples. Similar research with the use of a four-ball machine was performed in [37] to assess the impact of temperature, load, and concentration of the biodiesel upon wear and friction. In the works [38,39], the authors also used a four-ball tester to investigate the tribological performance of tyre pyrolysis oil. There have also been works concerning the use of four-ball machine to research the tribological characteristics of Calophyllum inophyllum (CI) biodiesel as a lubricity enhancer [40,41,42]. To compare lubrication properties in this study, the functionality of a four-ball machine, consisting of the possibility of determining the scuffing load, was used [43,44,45,46].

Previous studies regarding the lubricating properties of diesel fuel and ethanol mixtures have focused on comparing the WSD (wear scar diameter) parameter, determined based on tests carried out following the HFRR method [47,48]. For example, Kuszewski et al. [32] observed no significant differences in the value of the WSD parameter for diesel fuel and ethanol blends at different ethanol volumes. One reason for this was the gradual evaporation of ethanol, which is a natural consequence of the long test duration. As a result, the obtained results did not align with observations during engine tests concerning the wear of injection equipment elements with ethanol fueling [49]. Other studies, particularly [48,50], have shown that the HFFR method for low fuel additive concentrations is characterized by low sensitivity to lubricity results. Under such conditions, some polar compounds, due to their low concentrations, may not produce a sufficient lubrication film, which can lead to degradation in the tribological node [51]. It has also been shown that the dominant wear observed in the HFRR method is delamination and adhesive wear [32,52].

To address this challenge, this work proposes another lubricity assessment criterion, based on the measurement of the scuffing load with a four-ball machine, which, in the case of diesel–ethanol blends, reflects the ability to create a boundary lubricating layer. This result is possible due to the specificity of the test, which is characterized by a short duration. This is an alternative approach to measuring the lubricity criterion, which can be particularly dedicated to ethanol–diesel fuel blends. The advantage of the presented method is the short time of determination. Under the conditions of the standard assessing lubricity by HFRR method, a full view of the lubricity of blends of diesel fuel and ethanol is not obtained because the test lasts 75 min, which intensifies the evaporation of ethanol from the blend.

The purpose of this study was to determine the extent to which certain volume fractions of ethanol in a typical diesel fuel affect the lubricating properties of the blends. The lubrication criterion utilized in this study was the scuffing load. The tests were carried out using a four-ball machine, which provided a continuous increase of the load force of the tribosystem.

## 2. Experimental Setup and Methodology

**Figure 1:**
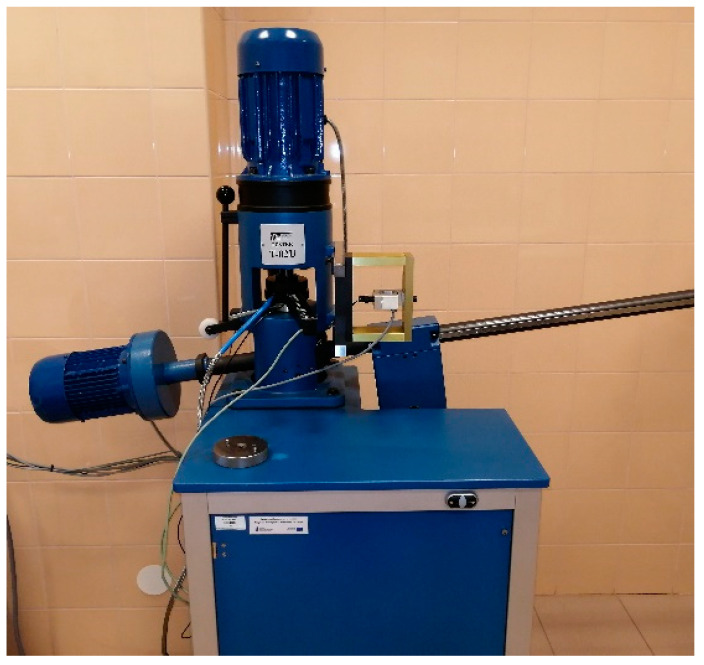
The four-ball machine T-02U used to determine the lubricity tests.

In this research, a four-ball T-02U machine (Figure 1) was used, which consists of a testing machine and measurement and control system.

**Figure 2:**
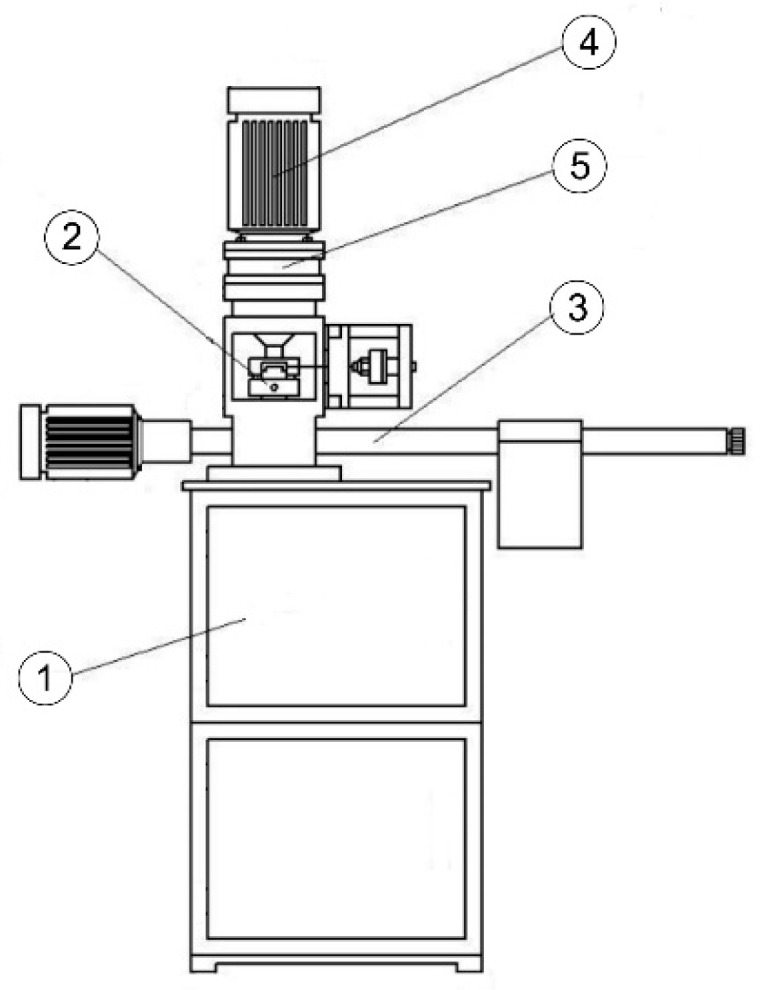
Four-ball machine T-02U [53]; 1, base; 2, ball holder; 3, loading unit of tribosystem; 4, drive unit; 5, body.

**Figure 3:**
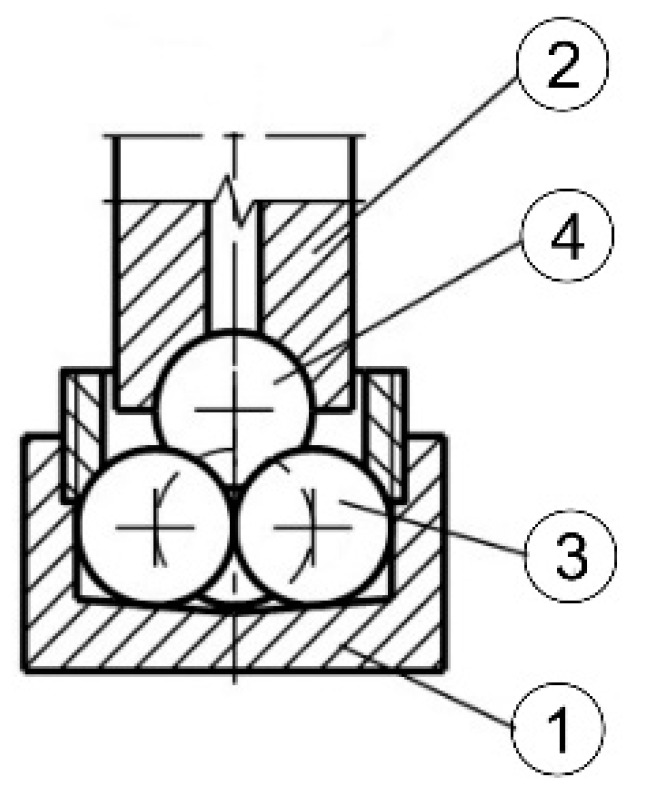
Four-ball tribosystem [53]; 1, ball pot; 2, ball chuck; 3, lower balls; 4, top ball.

The mechanical part of the machine (testing machine) consists of the body, drive unit, tribosystem loading unit, ball chuck, and base (Figure 2) [53]. Figure 3 shows the tribosystem, which consists of three stationary balls fixed in the ball pot that are pressed at the required load against the top ball. The top ball is fixed in the ball chuck, rotating at the defined speed.

The test elements were standardized bearing balls with a nominal 1/2” diameter made of bearing steel ŁH15 with a hardness of 60–65 HRC. The detailed parameters of the test balls are provided in Table 1.

Table 2 shows the technical data of the four-ball machine. The mechanical system enabled a linear increase of tribosystem load during the test run. The device was controlled using an asynchronous motor controller, microprocessor controller, and a computer with special control software.

Lubrication tests were carried out under conditions of continuously increasing load, similarly to authors’ previous studies [54,55]. The spindle rotational speed during the test run was 500 rpm, while the load build-up speed was 409 N/s. The initial temperature of the fuel sample at the beginning of the test run was 60 ± 4 °C; this is the temperature at which HFRR lubricity measurements were carried out. In the adopted test method, seizure of the tribosystem occurs when the limit value of the friction torque of 10 nM is reached. This value is determined by the mechanical durability of the upper ball chuck in the tribosystem. During the test, the course of the friction torque, M_T_, the course of linearly increasing load on the tribosystem, P, and the friction coefficient, µ, were recorded. The charts for these parameters, presented in the Discussion Section, were prepared for the measured data from 0.8 to 2.0 s of the test run. The initiation of scuffing of the tribosystem occurred for each sample within this time range. Since the course of load P change is a fixed parameter, it was approximated by a linear function. The values of scuffing load P were determined for the time from the start of the test run at which the first significant increase in friction coefficient and friction torque occurred. In the charts, these points were connected by a straight line. The intersection of this line with the course of the load P indicates the value of the force P_T_, which is the criterion for evaluating lubricity in the adopted method. Since the measuring system recorded data at a frequency of 75 Hz, in order to identify the point on the time axis which was assumed to be the initiation of scuffing, the number of measurement points taken into consideration and presented on the diagrams was limited to a frequency of 15 Hz. Based on the adopted methodology, and rounding the P_T_ load value to the tens of N, the measurement uncertainty was determined to be ±10 N.

The sample with the best lubricating properties was considered to be that for which the boundary layer showed the highest resistance to breaking, i.e., the sample with the highest value for the scuffing load. Detailed descriptions for determining the scuffing load are provided in [45,55,56,57].

## 3. Sample Characterization

Lubrication tests were carried out for eight fuel samples. One of these was a commercially available standard grade B diesel fuel, meeting the standard EN-PN 590 requirements. Additional samples included blends of standard diesel fuel with ethanol volume fractions ranging from 2% to 14%. These blends were prepared at the same temperature of diesel fuel and alcohol of 22 ± 1 °C. Due to the need to obtain homogeneity and stability in the blends, dehydrated ethyl alcohol with a purity of more than 99.5% was used. During the entire lubrication test cycle, the prepared diesel–ethanol blends were observed to be homogeneous. Fuel samples were stored in sealed glass vessels at 22 ± 1 °C. Descriptions of individual fuel samples are presented in Table 3. The values of the basic parameters of the fuel samples are listed in Table 4.

## 4. Discussion

**Figure 4:**
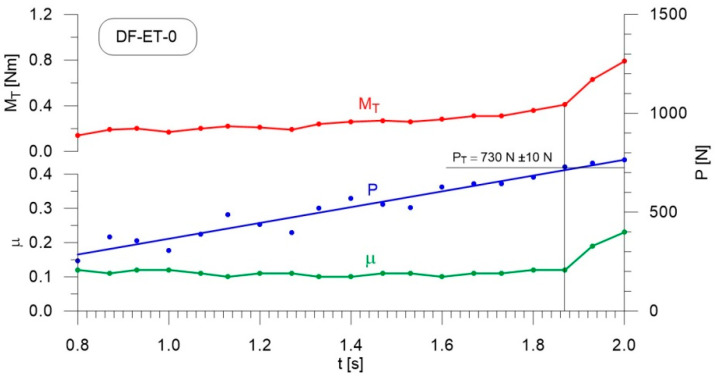
Courses of the load, P, the friction torque, M_T_, and the friction coefficient, µ, for diesel fuel without ethanol fraction.

**Figure 5:**
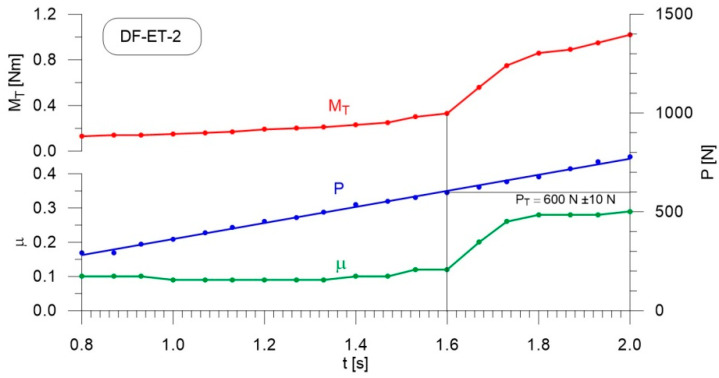
Courses of the load, P, the friction torque, M_T_, and the friction coefficient, µ, for diesel–ethanol blend with a 2% ethanol volume fraction.

**Figure 6:**
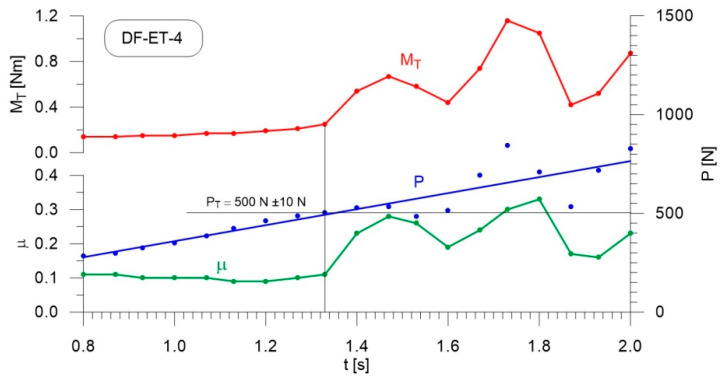
Courses of the load, P, the friction torque, M_T_, and the friction coefficient, µ for diesel–ethanol blend with a 4% ethanol volume fraction.

**Figure 7:**
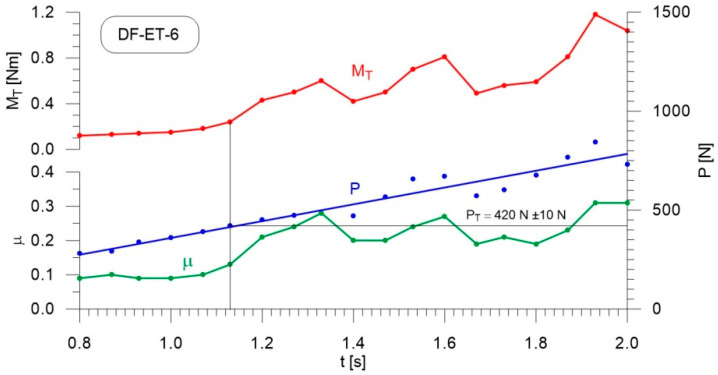
Courses of the load, P, the friction torque, M_T_, and the friction coefficient, µ, for diesel–ethanol blend with a 6% ethanol volume fraction.

**Figure 8:**
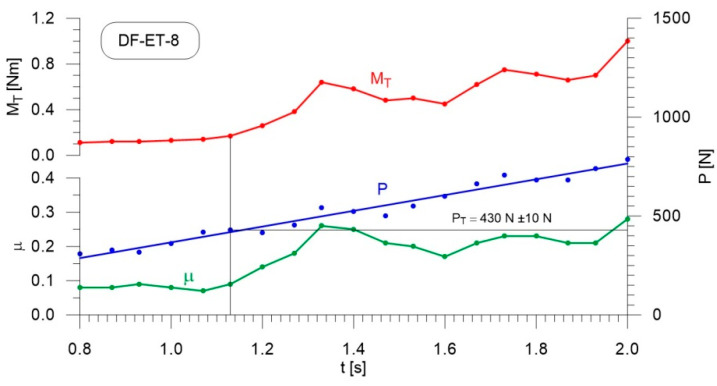
Courses of the load, P, the friction torque, M_T_, and the friction coefficient, µ, for diesel–ethanol blend with an 8% ethanol volume fraction.

**Figure 9:**
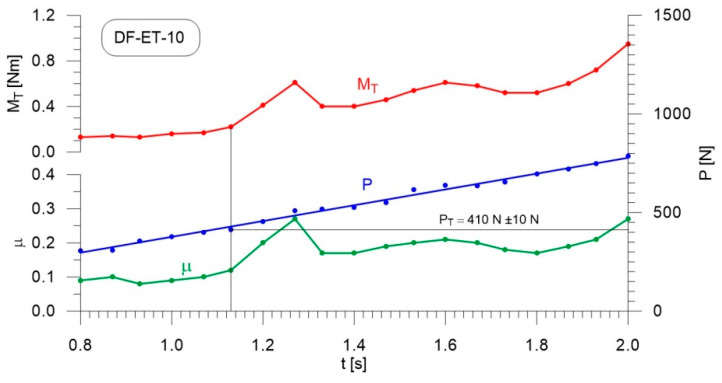
Courses of the load, P, the friction torque, M_T_, and the friction coefficient, µ, for diesel–ethanol blend with a 10% ethanol volume fraction.

**Figure 10:**
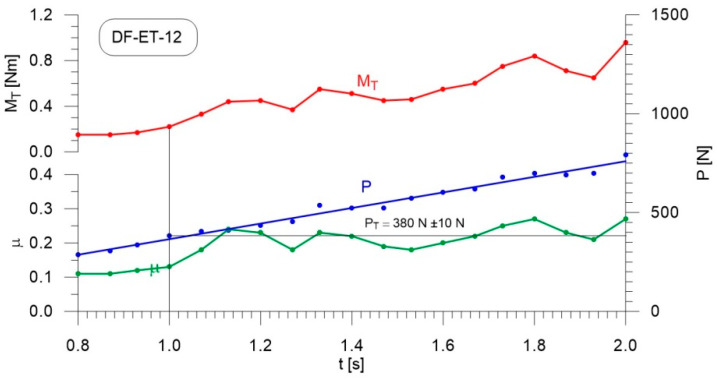
Courses of the load, P, the friction torque, M_T_, and the friction coefficient, µ, for diesel–ethanol blend with a 12% ethanol volume fraction.

**Figure 11:**
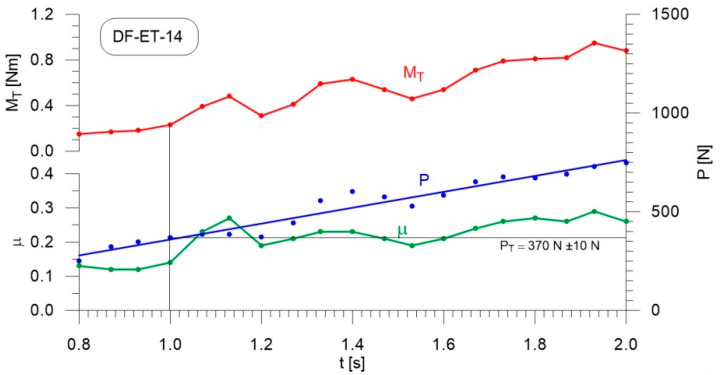
Courses of the load, P, the friction torque, M_T_, and the friction coefficient, µ, for diesel–ethanol blend with a 14% ethanol volume fraction.

Figure 4, Figure 5, Figure 6, Figure 7, Figure 8, Figure 9, Figure 10 and Figure 11 show the load courses of the tribosystem, P, the friction torque, M_T_, and the friction coefficient, µ, for the analyzed fuel samples. In accordance with the adopted criterion [57] for assessing the lubricity of individual samples, the diagrams also show the values of scuffing load, P_T_. The higher the value of this parameter, the more effective the lubricating properties. The diagrams were prepared using data for the first 2 s of the test run.

As can be seen from the figures, diesel fuel without ethanol showed the best lubricity properties among the fuel samples tested, with a recorded value for the scuffing load of 730 N (Figure 4). The increase in the friction coefficient and the friction torque occurred after 1.87 s from the beginning of the test. The increase in the volume fraction of ethanol in the ethanol–diesel fuel blends resulted in a decrease in the scuffing load of P_T_, i.e., deterioration of the lubricating properties. The lowest value of this parameter was recorded for DF-ET-14, at P_T_ = 370 N, after 1.00 s from the start of the test (Figure 11). At 2% ethanol volume fraction, P_T_ = 600 N was obtained after 1.60 s from the start of the test (Figure 5). Increasing the ethanol volume fraction to 4% resulted in a reduction of the value to P_T_ = 500 N after 1.36 s from the start of the test (Figure 6). Increasing the ethanol volume fraction further to 6% (Figure 7), led to a lower value of the scuffing load, P_T_ = 420 N being obtained after 1.13 s. Additional increases in the volume fraction of ethanol did not cause significant changes in the value of the scuffing load P_T_ and the time of its occurrence. For the ethanol volume fraction of 8% (Figure 8), P_T_ = 430 N, while 10% ethanol (Figure 9), P_T_ = 410 N; the time to occurrence of these scuffing load values was the same as in the case of the ethanol–diesel fuel blend containing 6% ethanol, i.e., approx. 1.13 s from the beginning of the test. Increasing the volume fraction of ethanol to 10% in the ethanol–diesel fuel blend led to a slight decrease to P_T_ = 380 N, after 1.00 s from the start of the test (Figure 10).

**Figure 12:**
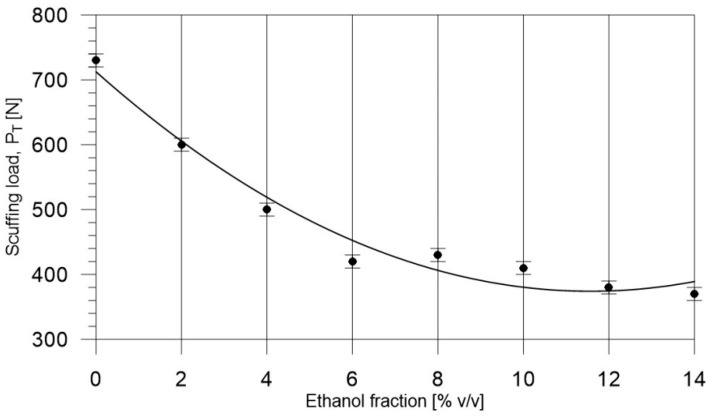
Effect of ethanol volume fraction in a diesel–ethanol blend on the scuffing load, P_T_.

From these measurements, the data presented in Figure 4, Figure 5, Figure 6, Figure 7, Figure 8, Figure 9, Figure 10 and Figure 11, and their comparisons listed in Table 5 and Figure 12, it can be seen that the lubricity of the blend deteriorated with increasing ethanol volume fraction in the ethanol–diesel fuel blends, as evidenced by the decrease in the value of the scuffing load. Under the conditions of the tests, the steepest drop in the scuffing load, P_T_, in relation to the DF-ET-0 sample, was noted for the highest ethanol volume fraction sample, DF-ET-14. Figure 12 also shows that starting from 6–14% ethanol volume fraction, the changes in the scuffing load, P_T_, were smaller than in the case of ethanol fractions from 0–6%.

A similar effect for the addition of ethanol on the lubricating properties was obtained by the authors of [59], who also carried out tests using a four-ball machine, and utilizing the diameter of the wear scar on the ball to measure lubricity. In these tests, a significant increase in the wear scar diameter was obtained for the ethanol volume fraction in the fuel blend of about 5%. The results confirmed the operation observations presented in the studies [25,60], that the increase in the ethanol fraction in diesel fuel causes deterioration of the blend lubricating properties. This leads to accelerated wear of the injection system parts.

## 5. Conclusions

The major findings of this study are as follows:

(1) Tests of lubricating properties carried out in accordance with the standard HFRR method for mixtures of diesel fuel and ethanol are associated with long test times, which lead to ethanol evaporation and changes in the composition of the tested fuel sample under elevated temperatures. Therefore, the authors conducted tests using a four-ball machine with a continuously increasing load. The obtained results provided additional insights into the influence of ethanol addition to diesel fuel on lubricating properties, while limiting the ethanol evaporation process.
(2) The presented results show that under the conditions of the lubrication tests carried out using a four-ball machine and assuming the value of the scuffing load as the lubrication criterion, an increase in the ethanol volume fraction in ethanol–diesel fuel blends resulted in the deterioration of the lubricating properties of the blend. In addition, a non-linear decrease in the scuffing load value was noted, as well as an increase in the ethanol volume fraction in the ethanol–diesel fuel blend.
(3) The results observed in this study do not correspond to the results of lubrication tests carried out using the HFRR method presented in [32]. In that study, for the same fuel samples, a negligible influence of ethanol fraction in ethanol–diesel blends on changes in the lubricity of the blend was noted. Discrepancies in the general conclusions are results of different test conditions and different lubricity assessment criteria.
(4) The HFRR method, which is suitable for testing the lubricating properties of diesel fuel, is not appropriate for diesel fuels with volatile additives such as ethanol.

Further work will be directed towards extending the presented method of comparative assessment of fuel lubricity, particularly in the area of identification of the point constituting the initiation of scuffing, WSD analysis, and SEM micrographs.

## Figures and Tables

**Table 1:** Test ball parameters.

**Steel grade**	ŁH15
**Diameter, inch**	1/2
**Roughness Ra, µm**	0.032
**Hardness, HRC**	60–65
**Chemical composition, %**	C: 0.95–1.10, Mn: 0.25–0.45, Si: 0.15–0.35, P: <0.027, S: <0.020, Cr: 1.30–1.65, Ni: <0.3, Cu: <0.25

**Table 2:** Technical data of the four-ball machine.

Specification	Units	Detail	Accuracy
Model	-	T-02U	-
Speed	RPM	300–1800	1
Sample temperature	°C	ambient temperature to 180	0.5
Maximum axial load	N	7850	0.5
Accuracy of motion resistance measurement	%	sensor Hottinger S2; 0–100 N	0.02
Accuracy of load measurement	%	force transducer Hottinger C9B; 0–10 kN	0.5

**Table 3:** Labels of the fuel samples.

Sample Label	Volume Fraction (%)	
	Diesel Fuel	Ethanol
DF-ET-0	100	0
DF-ET-2	98	2
DF-ET-4	96	4
DF-ET-6	94	6
DF-ET-8	92	8
DF-ET-10	90	10
DF-ET-12	88	12
DF-ET-14	86	14

**Table 4:** Properties of the ethanol–diesel fuel blends used in the testing.

Property	Method	Value							
		DF-ET-0	DF-ET-2	DF-ET-4	DF-ET-6	DF-ET-8	DF-ET-10	DF-ET-12	DF-ET-14
Derived cetane number (DCN)	ASTM D7668	55.2	50.3 ^1^	48.6 ^1^	47.4 ^1^	45.8 ^1^	45.4 ^1^	42.7 ^1^	41.3 ^1^
Higher heating value (MJ/kg)	PN-C-04375-3	45.97	45.42	44.90	44.71	44.36	43.98	43.68	43.30
Kinematic viscosity at 60 °C (mm^2^/s)	PN-EN ISO 3104	2.04	1.91	1.84	1.78	1.72	1.67	1.64	1.60
Dynamic viscosity at 60 °C (mPa·s)	PN-EN ISO 3104	1.64	1.53	1.47	1.42	1.37	1.33	1.30	1.27
Density at 60 °C (g/cm^3^)	PN-EN ISO 12185	0.803	0.801	0.799	0.798	0.797	0.796	0.795	0.793
Flash point (°C)	EN ISO 2719 A	65.5	-	-	-	-	-	-	-
Water content (mg/kg)	EN ISO 12937	23	112	179	259	343	427	498	587
CFPP (°C)	EN 116	−5	−7	−8	−7	−6	−7	−6	−6
Sulphur content (mg/kg)	PN-EN ISO 20846	5.2	-	-	-	-	-	-	-
Lubricity WSD (μm)	PN-EN ISO 12156(1)	189.5	196.5	188.0	188.5	197.0	180.5	184.5	193.0
FAME content (% *v*/*v*)	Infrared analysis (instrument TD PPA–PetroSpec by PAC)	6.70	6.57 ^2^	6.43 ^2^	6.30 ^2^	6.16 ^2^	6.03 ^2^	5.90 ^2^	5.76 ^2^

^1^—data provided by [58], ^2^—calculated.

**Table 5:** Lubrication test results for individual fuel samples.

Fuel Sample	Value of Scuffing Load P_T_ (N)	Percentage Decrease of Scuffing Load P_T_ Compared to Sample DF-ET-0 (%)	Initiation Time of the Scuffing from the Start of the Test Run (s)
DF-ET-0	730 ± 10	-	1.87
DF-ET-2	600 ± 10	18	1.60
DF-ET-4	500 ± 10	32	1.36
DF-ET-6	420 ± 10	42	1.13
DF-ET-8	430 ± 10	41	1.13
DF-ET-10	410 ± 10	44	1.13
DF-ET-12	380 ± 10	48	1.00
DF-ET-14	370 ± 10	49	1.00

## Data Availability

Data is contained within the article material.
